# Supplementary material for: Meta-Analysis and Gene Set Enrichment Relative to ER Status Reveal Elevated Activity of MYC and E2F in the “Basal” Breast Cancer Subgroup
Source: PLoS One. 2009 Mar 9;4(3):e4710. doi: 10.1371/journal.pone.0004710 (PMC2650420; doi:10.1371/journal.pone.0004710)
Supplement: Dataset S1 — GSEA results from first screen. The file contains a zipped archive of the HTML results of the second GSEA screen. Reports for enriched or depleted datasets may be accessed from the “index.html” link within the my_analysis.GseaPreranked.1219975950625.rpt.zip ZIP archive. Enrichment plots are available for each of the 50 most enriched and depleted gene sets. (13.32 MB ZIP) [file pone.0004710.s001.zip › my_analysis.GseaPreranked.1219975950625.rpt.zip!//SERUM_FIBROBLAST_CELLCYCLE.html]

Details for gene set SERUM\_FIBROBLAST\_CELLCYCLE[GSEA]

|  || Dataset | zSco.ERneg.to.ERpos\_collapsed |
| Phenotype | NoPhenotypeAvailable |
| Upregulated in class | na\_pos |
| GeneSet | SERUM\_FIBROBLAST\_CELLCYCLE |
| Enrichment Score (ES) | 0.70528877 |
| Normalized Enrichment Score (NES) | 2.9416814 |
| Nominal p-value | 0.0 |
| FDR q-value | 0.0 |
| FWER p-Value | 0.0 |
Table: GSEA Results Summary

  

Fig 1: Enrichment plot: SERUM\_FIBROBLAST\_CELLCYCLE      
 Profile of the Running ES Score & Positions of GeneSet Members on the Rank Ordered List

  

| PROBE | GENE SYMBOL | GENE\_TITLE | RANK IN GENE LIST | RANK METRIC SCORE | RUNNING ES | CORE ENRICHMENT || 1 | FOXM1 |  |  | 21 | 6.760 | 0.0166 | Yes |
| 2 | MCM5 |  |  | 31 | 6.533 | 0.0335 | Yes |
| 3 | BUB1 |  |  | 41 | 6.445 | 0.0501 | Yes |
| 4 | CENPA |  |  | 51 | 6.315 | 0.0665 | Yes |
| 5 | CCNA2 |  |  | 52 | 6.297 | 0.0834 | Yes |
| 6 | DKFZP762E1312 |  |  | 61 | 6.161 | 0.0994 | Yes |
| 7 | ANP32E |  |  | 84 | 5.988 | 0.1138 | Yes |
| 8 | TRIP13 |  |  | 96 | 5.890 | 0.1288 | Yes |
| 9 | FANCA |  |  | 99 | 5.866 | 0.1444 | Yes |
| 10 | GTSE1 |  |  | 129 | 5.694 | 0.1576 | Yes |
| 11 | FAM64A |  |  | 137 | 5.658 | 0.1723 | Yes |
| 12 | KIFC1 |  |  | 156 | 5.525 | 0.1858 | Yes |
| 13 | MCM4 |  |  | 180 | 5.436 | 0.1986 | Yes |
| 14 | MELK |  |  | 181 | 5.424 | 0.2132 | Yes |
| 15 | CDCA8 |  |  | 194 | 5.328 | 0.2267 | Yes |
| 16 | MCM6 |  |  | 198 | 5.317 | 0.2407 | Yes |
| 17 | GMNN |  |  | 206 | 5.302 | 0.2545 | Yes |
| 18 | EXO1 |  |  | 218 | 5.239 | 0.2677 | Yes |
| 19 | USP1 |  |  | 222 | 5.212 | 0.2815 | Yes |
| 20 | PTTG1 |  |  | 225 | 5.206 | 0.2954 | Yes |
| 21 | TPX2 |  |  | 231 | 5.180 | 0.3089 | Yes |
| 22 | CKS2 |  |  | 239 | 5.145 | 0.3222 | Yes |
| 23 | RAD51AP1 |  |  | 267 | 5.028 | 0.3337 | Yes |
| 24 | CENPF |  |  | 268 | 5.028 | 0.3472 | Yes |
| 25 | CDC25A |  |  | 281 | 4.993 | 0.3598 | Yes |
| 26 | EZH2 |  |  | 312 | 4.888 | 0.3707 | Yes |
| 27 | NCAPH |  |  | 348 | 4.799 | 0.3809 | Yes |
| 28 | RFC4 |  |  | 370 | 4.741 | 0.3921 | Yes |
| 29 | CKS1B |  |  | 416 | 4.638 | 0.4011 | Yes |
| 30 | CCNB2 |  |  | 431 | 4.589 | 0.4124 | Yes |
| 31 | SMC4 |  |  | 448 | 4.519 | 0.4234 | Yes |
| 32 | AURKA |  |  | 460 | 4.483 | 0.4346 | Yes |
| 33 | MAD2L1 |  |  | 477 | 4.446 | 0.4454 | Yes |
| 34 | PSRC1 |  |  | 493 | 4.363 | 0.4560 | Yes |
| 35 | DLG7 |  |  | 521 | 4.306 | 0.4655 | Yes |
| 36 | TUBA1 |  |  | 563 | 4.214 | 0.4737 | Yes |
| 37 | TUBB |  |  | 582 | 4.183 | 0.4836 | Yes |
| 38 | GINS3 |  |  | 637 | 4.059 | 0.4904 | Yes |
| 39 | AMD1 |  |  | 658 | 4.018 | 0.4997 | Yes |
| 40 | ADAMTS1 |  |  | 659 | 4.017 | 0.5105 | Yes |
| 41 | NCAPD3 |  |  | 661 | 4.016 | 0.5213 | Yes |
| 42 | TACC3 |  |  | 669 | 3.995 | 0.5315 | Yes |
| 43 | RANGAP1 |  |  | 712 | 3.909 | 0.5388 | Yes |
| 44 | CCDC99 |  |  | 737 | 3.863 | 0.5474 | Yes |
| 45 | FEN1 |  |  | 754 | 3.838 | 0.5565 | Yes |
| 46 | RFC2 |  |  | 762 | 3.821 | 0.5663 | Yes |
| 47 | MPHOSPH1 |  |  | 778 | 3.799 | 0.5754 | Yes |
| 48 | TYMS |  |  | 829 | 3.738 | 0.5816 | Yes |
| 49 | H2AFX |  |  | 930 | 3.591 | 0.5837 | Yes |
| 50 | PRIM2A |  |  | 941 | 3.564 | 0.5925 | Yes |
| 51 | PPIH |  |  | 943 | 3.562 | 0.6021 | Yes |
| 52 | DONSON |  |  | 944 | 3.561 | 0.6116 | Yes |
| 53 | PLK1 |  |  | 988 | 3.499 | 0.6178 | Yes |
| 54 | UBE2C |  |  | 994 | 3.489 | 0.6268 | Yes |
| 55 | KIF23 |  |  | 1030 | 3.442 | 0.6334 | Yes |
| 56 | CIITA |  |  | 1216 | 3.220 | 0.6281 | Yes |
| 57 | CDC2 |  |  | 1259 | 3.182 | 0.6335 | Yes |
| 58 | ASF1B |  |  | 1319 | 3.119 | 0.6374 | Yes |
| 59 | PCNA |  |  | 1346 | 3.087 | 0.6437 | Yes |
| 60 | HELLS |  |  | 1382 | 3.057 | 0.6493 | Yes |
| 61 | CENPM |  |  | 1453 | 2.995 | 0.6520 | Yes |
| 62 | CKAP2 |  |  | 1492 | 2.951 | 0.6571 | Yes |
| 63 | ATAD2 |  |  | 1532 | 2.917 | 0.6620 | Yes |
| 64 | HMMR |  |  | 1608 | 2.843 | 0.6640 | Yes |
| 65 | ILF2 |  |  | 1644 | 2.807 | 0.6689 | Yes |
| 66 | KIAA1794 |  |  | 1724 | 2.747 | 0.6703 | Yes |
| 67 | CDC25C |  |  | 1794 | 2.700 | 0.6723 | Yes |
| 68 | PWP1 |  |  | 1799 | 2.692 | 0.6793 | Yes |
| 69 | CDKN3 |  |  | 1827 | 2.674 | 0.6844 | Yes |
| 70 | PBK |  |  | 1853 | 2.647 | 0.6896 | Yes |
| 71 | TIPIN |  |  | 1854 | 2.646 | 0.6968 | Yes |
| 72 | CENPQ |  |  | 1908 | 2.601 | 0.6997 | Yes |
| 73 | ITGB3 |  |  | 2000 | 2.531 | 0.6997 | Yes |
| 74 | RRM2 |  |  | 2047 | 2.485 | 0.7029 | Yes |
| 75 | PHTF2 |  |  | 2148 | 2.418 | 0.7018 | Yes |
| 76 | MET |  |  | 2188 | 2.394 | 0.7053 | Yes |
| 77 | FANCG |  |  | 2282 | 2.332 | 0.7045 | No |
| 78 | LMNB1 |  |  | 2764 | 2.060 | 0.6737 | No |
| 79 | BARD1 |  |  | 3028 | 1.920 | 0.6589 | No |
| 80 | CDC6 |  |  | 3185 | 1.847 | 0.6521 | No |
| 81 | CCNF |  |  | 3265 | 1.809 | 0.6510 | No |
| 82 | PAQR4 |  |  | 3434 | 1.736 | 0.6429 | No |
| 83 | KNTC1 |  |  | 3562 | 1.679 | 0.6378 | No |
| 84 | TOP2A |  |  | 3735 | 1.610 | 0.6291 | No |
| 85 | TUBB2C |  |  | 3736 | 1.610 | 0.6335 | No |
| 86 | NALP2 |  |  | 4369 | 1.367 | 0.5893 | No |
| 87 | KIAA0101 |  |  | 4770 | 1.230 | 0.5623 | No |
| 88 | RRM1 |  |  | 4872 | 1.197 | 0.5579 | No |
| 89 | HN1 |  |  | 4953 | 1.171 | 0.5550 | No |
| 90 | RECQL4 |  |  | 5037 | 1.141 | 0.5518 | No |
| 91 | MLF1IP |  |  | 5991 | 0.815 | 0.4818 | No |
| 92 | SPAG5 |  |  | 6030 | 0.800 | 0.4811 | No |
| 93 | WSB1 |  |  | 6185 | 0.754 | 0.4715 | No |
| 94 | PRIM1 |  |  | 6525 | 0.642 | 0.4476 | No |
| 95 | SCML1 |  |  | 6615 | 0.617 | 0.4425 | No |
| 96 | SGCD |  |  | 6831 | 0.541 | 0.4277 | No |
| 97 | CDKL5 |  |  | 7173 | 0.426 | 0.4030 | No |
| 98 | CTNNA1 |  |  | 8309 | -0.001 | 0.3171 | No |
| 99 | YWHAH |  |  | 8629 | -0.142 | 0.2933 | No |
| 100 | ANKRD10 |  |  | 8942 | -0.282 | 0.2705 | No |
| 101 | MAPK13 |  |  | 9238 | -0.422 | 0.2493 | No |
| 102 | SDC1 |  |  | 9416 | -0.521 | 0.2373 | No |
| 103 | GINS2 |  |  | 9500 | -0.560 | 0.2325 | No |
| 104 | CASP3 |  |  | 9899 | -0.783 | 0.2045 | No |
| 105 | TIMP1 |  |  | 11230 | -1.769 | 0.1086 | No |
| 106 | H2-ALPHA |  |  | 11262 | -1.794 | 0.1110 | No |
| 107 | ABCC5 |  |  | 12662 | -3.546 | 0.0147 | No |
| 108 | EFHC1 |  |  | 12825 | -3.941 | 0.0130 | No |
| 109 | HIST1H2AC |  |  | 12865 | -4.048 | 0.0210 | No |
| 110 | TNCRNA |  |  | 13139 | -4.984 | 0.0137 | No |
Table: GSEA details [plain text format]

  

Fig 2: SERUM\_FIBROBLAST\_CELLCYCLE: Random ES distribution      
 Gene set null distribution of ES for **SERUM\_FIBROBLAST\_CELLCYCLE**
